# DNA Data Visualization (DDV): Software for Generating Web-Based Interfaces Supporting Navigation and Analysis of DNA Sequence Data of Entire Genomes

**DOI:** 10.1371/journal.pone.0143615

**Published:** 2015-12-04

**Authors:** Tomasz Neugebauer, Eric Bordeleau, Vincent Burrus, Ryszard Brzezinski

**Affiliations:** 1 Concordia University Libraries, Montreal, Quebec, Canada; 2 Département de Biologie, Faculté des Sciences, Université de Sherbrooke, Sherbrooke, Quebec, Canada; University of Helsinki, FINLAND

## Abstract

Data visualization methods are necessary during the exploration and analysis activities of an increasingly data-intensive scientific process. There are few existing visualization methods for raw nucleotide sequences of a whole genome or chromosome. Software for data visualization should allow the researchers to create accessible data visualization interfaces that can be exported and shared with others on the web. Herein, novel software developed for generating DNA data visualization interfaces is described. The software converts DNA data sets into images that are further processed as multi-scale images to be accessed through a web-based interface that supports zooming, panning and sequence fragment selection. Nucleotide composition frequencies and GC skew of a selected sequence segment can be obtained through the interface. The software was used to generate DNA data visualization of human and bacterial chromosomes. Examples of visually detectable features such as short and long direct repeats, long terminal repeats, mobile genetic elements, heterochromatic segments in microbial and human chromosomes, are presented. The software and its source code are available for download and further development. The visualization interfaces generated with the software allow for the immediate identification and observation of several types of sequence patterns in genomes of various sizes and origins. The visualization interfaces generated with the software are readily accessible through a web browser. This software is a useful research and teaching tool for genetics and structural genomics.

## Introduction

Information visualization can amplify cognition by storing massive amounts of information in quickly accessible forms and using visual representations to enhance the detection of patterns [1].Visualization of genomic data augments reasoning and analysis by facilitating the complementing of computational methods with human interpretation [2]. Graphical representations of genomic data allow for rapid viewing and identification of characteristics of specific regions of the genome and new encoding patterns with biological significance.

In this paper, we present software that generates interactive, graphical representations of large nucleotide sequence data sets, accessible through an Internet browser. The method is scalable to large chromosomes such as those of *Homo sapiens* and generally speaking, the eukaryotic organisms.

Early attempts to visualize whole genome nucleotide sequences include the one presented by Makino *et al*. [3], who used printed representations on A4 paper to visualize complete nucleotide sequences. Makino *et al*. suggested that the regions in genomes that encode genes are more purine-rich than non-coding sequences. Thus, given their choice of red for A and black for G, Makino *et al*. stipulated that the “reddish/blackish stripes” that were visible on their printed visualization of the genomic sequence of *Mycoplasma pneumoniae* represent putative gene coding regions.

Yoshida *et al*. [4] chose the same four colors for visualizing nucleotides: blue for G, green for C, yellow for T and red for A. Yoshida *et al*. used visualization columns of variable width to locate tandem repeats in the *Escherichia coli* genome.

Seaman & Sanford visualization tool, Skittle [5], is a significant achievement and its literature review offers a critique of other prior efforts such as the DNA Rainbow project [6]. The DNA Rainbow project does not visualize the DNA data using the conventional FASTA data format consisting of less than 80 nucleotides per column; a large image without columns is used instead. The DNA Rainbow images are so large that they are practically difficult if not impossible to view and navigate using conventional technology such as a web browser. Skittle software is designed for a single user interacting with raw nucleotide data visualized with colors. Initially, the use of Skittle required downloading and installing the software on a desktop computer, with no option of exporting the visualization results to the web for collaboration and shared spaces. Skittle now also includes a beta of a web-based version of the application [7]. The implementation and approach to the visualization of the data is different in DDV from Skittle. Among notable differences, Skittle generates a single-column image based on portions of the data requested by the user, whereas DDV pre-generates all of the images required for visualizing the entire DNA data sequence as a multi-column, smoothly zoomable multi-scale image. The multi-column geometry of the visualizations makes it easier for users to visually parse long sequences of data while the use of multi-scale images allows for the integration of community supported web-based viewer technology.

The DNA Data Visualization (DDV) software described in this paper allows a user of a local desktop computer to generate visualization interfaces that can be exported to the web, increasing access and the potential for collaboration around the visualized data. Extending visualization tool functionality to support collaboration increases the scope and applicability of the visualizations. The method we propose in this work pre-generates all of the necessary images and offers an efficient way of navigating these images that display the genome at various levels of detail. It allows for the visualization of long raw nucleotide sequences through a display format that is optimized for access and interaction through the web. The zoom pyramid structure of pre-generated images in our method allows for smooth interaction and browsing of the data. The only requirements for accessing prepared visualizations are Internet access and a modern web browser. DDV includes functions for GC Skew plotting and for computing nucleotide composition density. The web output format of DDV leverages modular tools for biological data visualization and high-resolution image navigation. The functionality of easy selection of sequence fragments allows researchers to continue their analysis using tools external to DDV. The method creates visualizations that allow for the combination of seamless graphical human inspection and automated computation, an approach that is particularly effective.

## Materials and Methods

### Visualization method summary

**Fig 1:**
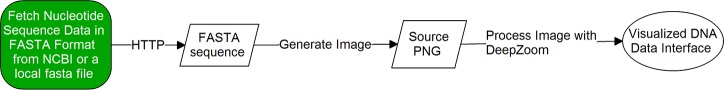
Summary of the visualization method. A source PNG image is generated to represent FASTA sequences. These source PNG images are then processed, including the creation of a Deep Zoom image pyramid. The resultant visualized interface includes scripts that compute nucleotide composition density and generate a GC skew graph.

**Fig 2:**
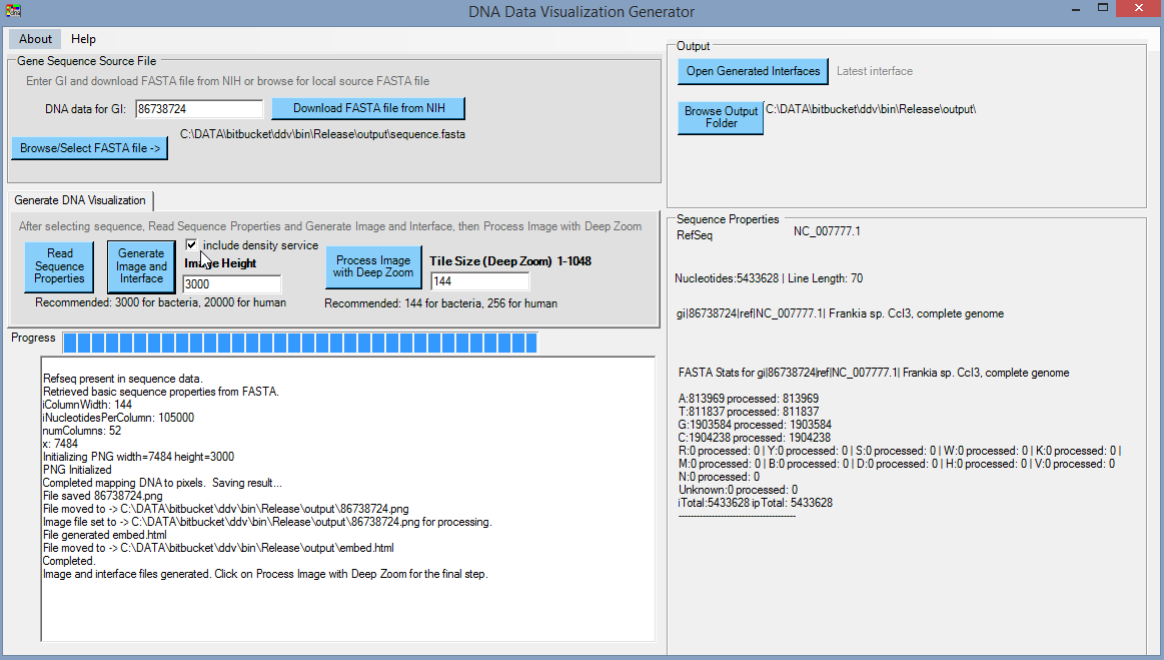
Screenshot of DNA Data Visualization generator (DDV) after it generates the source PNG for Frankia sp. CcI3 chromosome [GenBank: NC_007777]. User downloads/selects FASTA data file, selects the image height, and generates the source PNG image. The final step is to click on “Process Image with Deep Zoom” to complete the generation of a visualization interface for this dataset.

**Fig 3:**
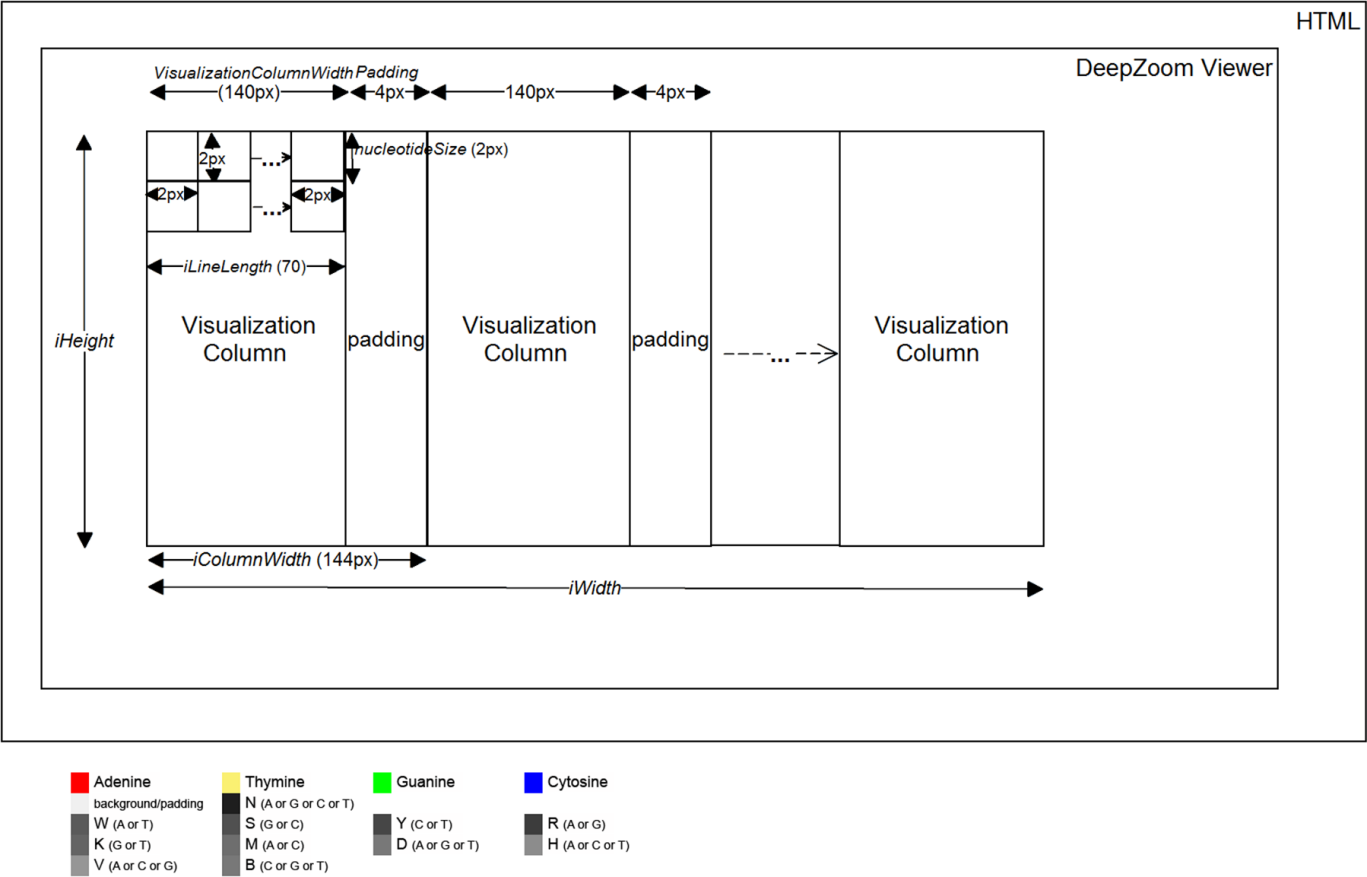
Source PNG image design. Each nucleotide is 2px X 2px, with 70 nucleotides per line. The height (iHeight) is set to 3000 px for bacterial genomes, and the value can be increased for larger data sets. The total width (iWidth) depends on length of the data. Each visualization column is separated by 4px of grey padding.

**Fig 4:**
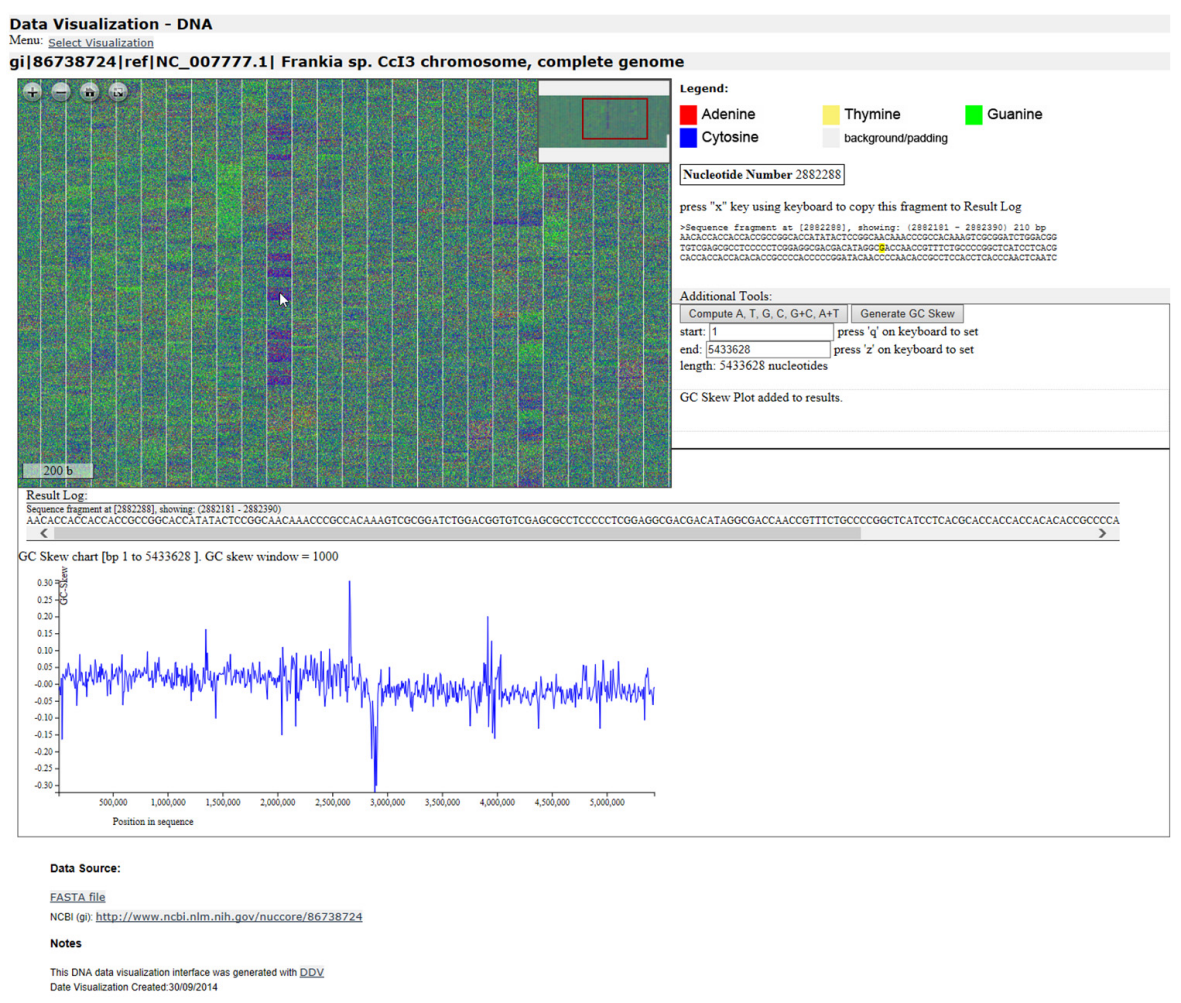
Screenshot of generated interface for visualized data set: *Frankia* sp. CcI3 genome [GenBank:NC_007777], 5433628 bp. Screenshot taken after computing nucleotide density for the whole sequence, zooming in, generating GC-Skew plot for the sequence, and selecting a sequence fragment at bp 2882288. The interface includes a scale bar (bottom left of Deep Zoom viewer) shown in bp units, and a viewport navigator (top right of Deep Zoom viewer) that shows zoom position and can also be used for panning.

The method is summarized in Fig 1. The first step in the visualization is to download the FASTA formatted nucleotide sequence. A screenshot of the DNA Data Visualization generator (DDV) during operation is shown in Fig 2. There are buttons on the DDV interface for downloading sequence data from National Center for Biotechnology Information (NCBI)’s nucleotide database, given a GI number as input, or the specification of a local FASTA file downloaded manually from another source. These nucleotide sequence data are then processed by clicking on buttons on the interface generator that correspond to the main steps in the algorithm for generating the visualization:

1. Read Sequence Properties: Read name, length and sequence identifiers such as RefSeq and GI numbers,
2. Generate Image and Interface:
  - Initialize an RGB bitmap of appropriate size for the visualization–this depends on the sequence size.
  - Process all of the nucleotides in the sequence by painting colors for each nucleotide on the image, generating a master image of large size. The design of this image is illustrated in Fig 3.
3. Process Image with Deep Zoom:
  - Generate the Deep Zoom Images (DZI) pyramid and XML from the master image using DeepZoomTools.dll [5].
  - Generate the HTML, CSS and JavaScript files for the completed DNA Data Visualization interface (Fig 4).

### DNA Data Interface Navigation

The end user interacts with the generated visualization through a web browser, using the navigation buttons (zoom in, zoom out, full screen, home) in the top left corner or the visual navigator in the top right corner. A screenshot example of a generated DNA data set interface for a bacterial genome [GenBank: NC_007777] is presented in Fig 4. The user can zoom in and out with the mouse wheel or navigation buttons. A scale bar on the bottom left shows length in bp units while a viewport navigator shows zoom position and can also be used for panning.

While pointing at a particular nucleotide on the visualization with the mouse pointer, the surrounding 210 bp sequence fragment is displayed as text on the interface. This sequence fragment can also be copied to the results by pressing the “x” key. Pressing the ‘q’ and ‘z’ keys on the keyboard marks the beginning and end of the portion of the sequence that can be sent for computation of % G+C and for the determination of the respective frequencies of the four nucleotides. The end user can also request a GC Skew plot for the sequence.

### Implementation Details

**Fig 5:**
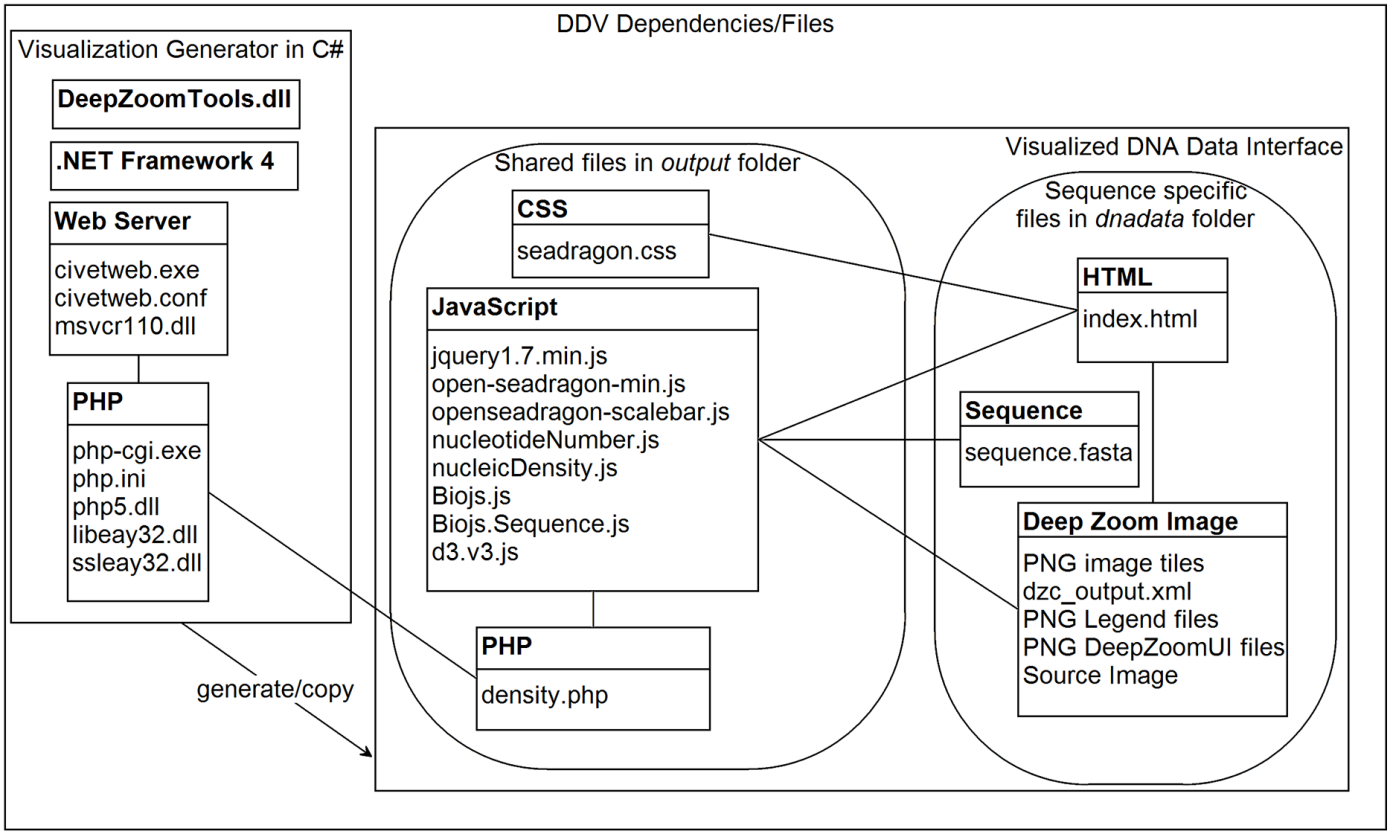
DDV generator dependencies and visualized DNA Data interface files. DDV is a C# application that generates sequence specific files for each DNA dataset, as well as the shared CSS, JavaScript, and PHP files in the output folder. The included civetweb serves the PHP files when the user is viewing the visualized interface on a local PC.

The full implementation is summarized in Fig 5. The DNA Data Visualization generator (DDV) is implemented in Visual Studio C# and compiled with. Net Framework 4. This application and all of its dependencies are available for download. Running DDV requires an operating system that supports. NET Framework 4, such as Windows Vista, Windows 7 or 8. The generated visualizations can be viewed on any operating system that supports a web browser with JavaScript. During initialization, DDV attempts to check if the correct version of. NET is installed, displaying a message directing the user to the free web installer [8] when necessary. DDV uses the Microsoft DeepZoomTools.dll in step 3.1 to generate the image tiles. DDV-generated visualizations are placed under the output folder which can then be placed on a web server for sharing and collaboration. DDV places all of the shared files into the root “output” folder, and the sequence-specific files in subfolders under “dnadata”.

Navigation of the deep zoom image is implemented with the open source OpenSeadragon JavaScript library [9]. OpenSeadragon has an active development community and features compatibility with desktop and mobile devices. In addition, BioJS [10] is used for sequence fragment display and D3.js [11] for GC Skew visualization. DDV also includes the minimal Civetweb web server [12] with PHP [13], ensuring that the generated visualizations are able to use PHP to compute nucleotide composition density from a Windows desktop computer as well as a hosted web server when the visualizations are placed online.

The images generated in step 2 are very large, as they contain the information from the entire chromosome in one image. For example, the image for *Clostridium difficile* 630 [GenBank: NC_009089] bacterial chromosome is 5 900px X 3 000px while the representation of the longest chromosome 1 [GenBank: NC_000003] of *H*. *sapiens* is 50 972px X 20 000px. Such large images would be difficult to display on the web without further processing. The geometric dimensions of these images and the colors used to represent the nucleotides are shown in Fig 3. The three additive primary colors: red, green, blue and the fourth color, yellow, are used to represent the four nucleotides. Light grey is used as the background color, black and dark shades of grey are used to represent various possible coded ‘unknowns’ in the FASTA format: N, W, S, Y, R, K, M, D, H, V, B. The legend on generated visualizations shows only those unknowns that actually appear in the sequence.

In Step 3, for bacterial chromosomes, tile size of 144px is selected so that at 1:1 level of magnification, each tile contains approximately 5 040 contiguous nucleotides (70 nucleotides/line × 72 lines = 5 040 nucleotides). The image pyramid is used by the web interface to offer the multiple views at various levels of magnification. This step creates thousands of small tiles at various levels of magnification which are loaded on demand while the user navigates the image. The *C*. *difficile* 630 [GenBank: NC_009089] bacterial chromosome (4 290 252 nt) is mapped to a source PNG image with dimensions 5 900px by 3 000px, which results in a Deep Zoom Image with 15 levels of magnification, ranging from 1 tile in levels 0 to 8, and gradually increasing to 861 tiles arranged in 41 columns by 21 rows at the highest level 14. In total, when users are navigating the *C*. *difficile* 630 visualization, they are actually navigating 1 195 image tiles. The second example, *H*. *sapiens* chromosome 1 [GenBank: NC_000003], the largest DNA molecule to which the method has been applied so far (247 249 719 nt) has the source image dimensions of 50 972px by 20 000px. Due to its much larger size, a tile size of 256px was adopted. These parameters result in a Deep Zoom folder/image structure with 17 levels of magnification, ranging from 1 image tile in levels 0 to 8, increasing to 15 800 image tiles arranged in 200 columns by 79 rows at the highest level 16. Therefore, when users are navigating the human chromosome 1 visualization, they are actually navigating a total of 21 155 image tiles.

In Step 3.2, the nucleotideNumber.js JavaScript converts the position currently pointed by the user on the Deep Zoom viewer into the corresponding nucleotide number on the sequence. This is computed based on properties of the coordinate system of Deep Zoom viewport as well as the geometry of the source PNG image (Fig 3), using the following formula:

$$Nucleotide=iLineLength\times \left(\frac{\text{iWidth}}{\text{nucleotideSize}}\right)\times \left\lfloor \frac{\text{x}\;\times \;\text{iWidth}}{\text{nucleotideSize}}\right\rfloor +iLineLength\times \left\lfloor \frac{\text{y}\;\times \;\text{iWidth}}{\text{nucleotideSize}}\right\rfloor +\left\lfloor \frac{(\text{x}\;\times \;\text{iWidth})\;\text{mod ColumnWidth}}{\text{nucleotideSize}}\right\rfloor +1$$

where iWidth is the width of the source PNG (pixels), iHeight is the height of the source PNG (pixels), nucleotideSize is the width of 1 nucleotide square on the source image (2 pixels), iLineLength is the number of nucleotides per line in column (70 nucleotides), VisualizationColumnWidth is the width of one visualization column (140 pixels), Padding is the separation between visualization columns (4 pixels), ColumnWidth is the sum of VisualizationColumnWidth and Padding (144 pixels), while (*x*, *y*) are the coordinates of the cursor position on the page minus the position of the viewer. In addition, the user is pointing at a nucleotide on the image as opposed to background or padding if and only if the following four conditions are true:

$$\left\{\begin{array}{l} 0<x<1\\ 0<y<\left(\frac{iHeight}{iWidth}\right)\\ Nucleotide\leq totalNucleotides\\ VisualizationColumnWidth\leq ((x\times iWidth)\;\mathrm{mod}ColumnWidth)\leq ColumnWidth \end{array}\right\}$$

where totalNucleotides is the total number of nucleotides in the sequence.

#### %G+C nucleic acid composition computation

The generated DNA Data Visualization Interface includes a PHP implemented function for computing %G+C and exact nucleic acid composition density of a selected portion of the visualized sequence, and to displays the computed results on the interface.

#### GC Skew Graph

The generated DNA Data Visualization Interface includes a JavaScript function for computing GC Skew data and plotting it on the interface using D3.js [11] library. The skew window is set depending on the number of nucleotides in the sequence, ranging from 50 for sequences of less than 100 000 nucleotides to 10 000 for sequences longer than 10 000 000 nucleotides.

#### Partial sequence data display and select

The generated DNA Data Visualization Interface includes the ability to display, select and copy 210 bp portions of the sequence currently pointed at on the interface. This is implemented with the help of BioJS [10]. A simple copy-paste operation allows for sending the selected sequence to external web services for further analysis.

#### Relative scale bar display

The generated visualizations also leverage OpenSeadragon’s scale bar. The bar is customized so that it shows the relationship between width on the image and the number of nucleotides, as the user zooms in and out.

## Results and Discussion

**Fig 6:**
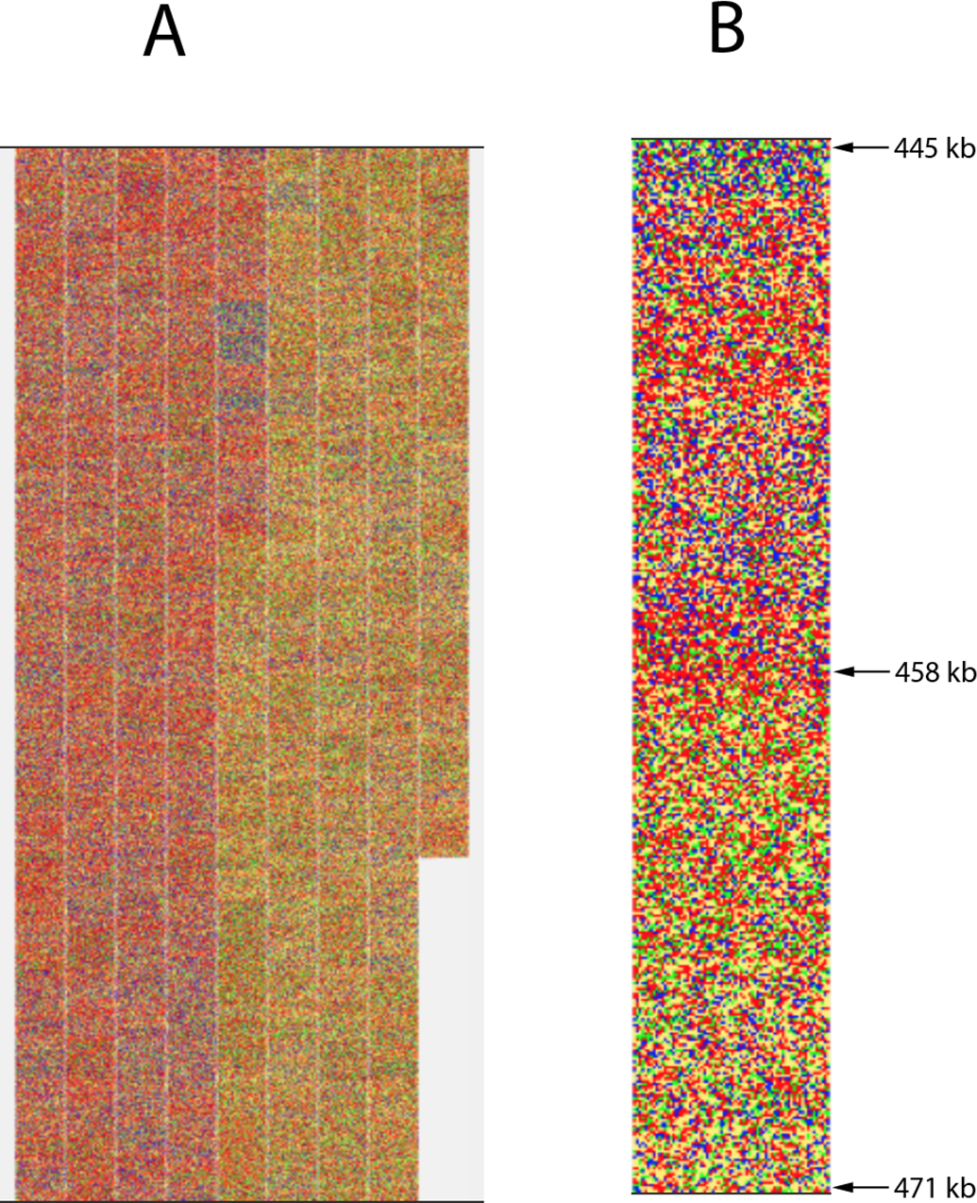
Visualization of the major linear chromosome of *Borrelia burgdorferi* B31 [GenBank: NC_001318]. A) Whole linear chromosome of 910724 bp; B) enlargement of the segment surrounding the origin of replication (localized between coordinates 458036 and 458227). Arrows indicate the distance from the beginning of the chromosome sequence.

**Fig 7:**
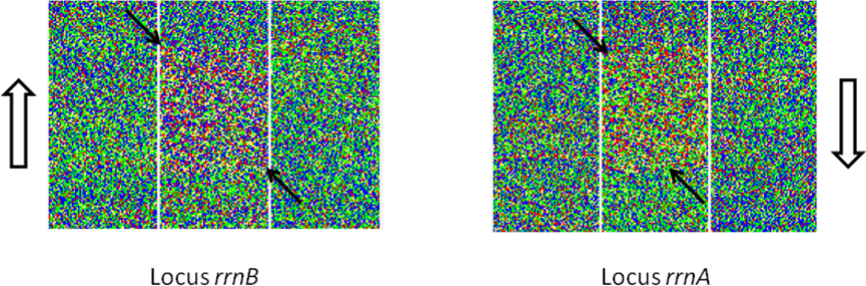
Ribosomal RNA gene clusters. Zoomed-in fragments of the visualization of the *Streptomyces coelicolor* A3(2) [GenBank: NC_003888] linear chromosome showing the *rrnB* and *rrnA* ribosomal gene clusters (coordinates 1916451 to 1921599 and 4530650 to 4535576). Arrows indicate the limits of each cluster. Empty arrows indicate the direction of chromosomal DNA replication fork movement and the direction of rRNA genes transcription.

**Fig 8:**
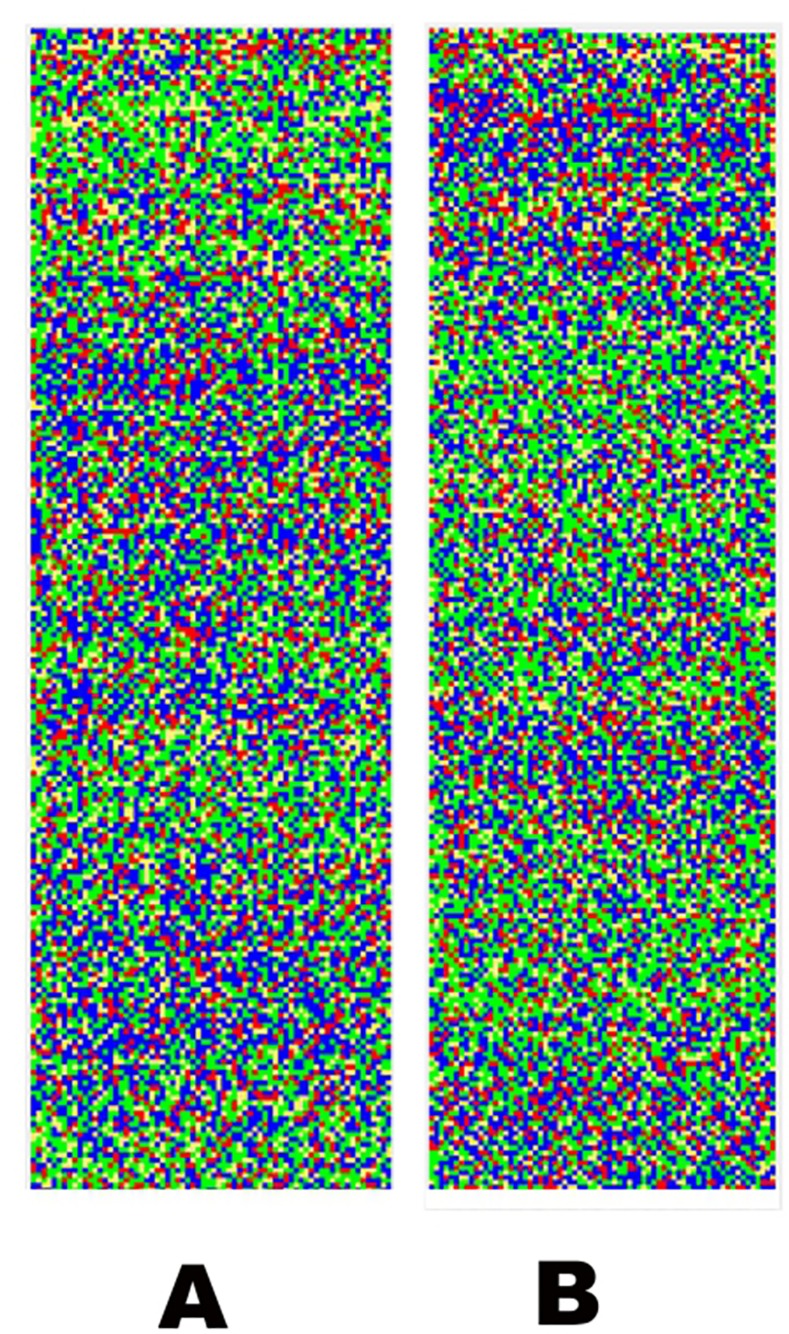
Visualization of the 16.5 kb-terminal segments of the *Streptomyces davawensis* JCM 4913 chromosome [GenBank: HE971709]. The segments are parts of the 33.3 kb LTIRs of this genome. A: image of the left end of the chromosome; B: 180°-rotated image of the right end of the chromosome.

**Fig 9:**
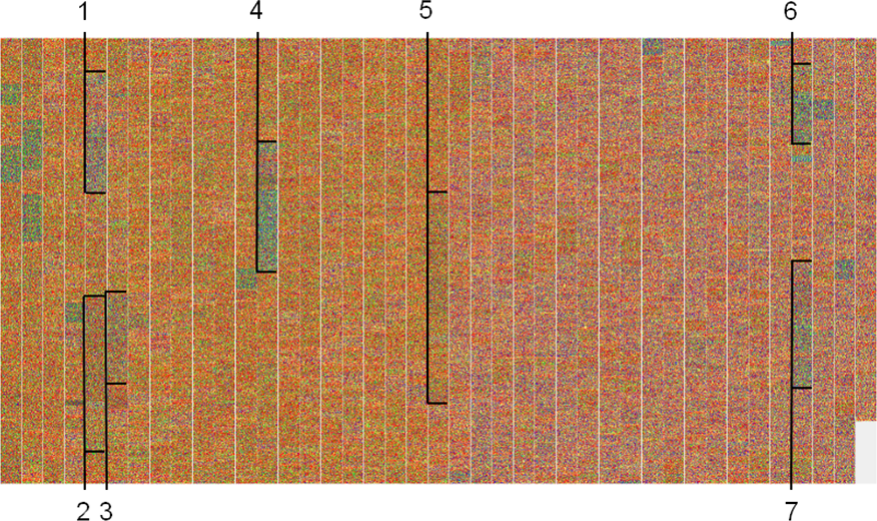
Visualization of the *Clostridium difficile* 630 chromosome [GenBank: NC_009089]. Segments corresponding to the seven known conjugative transposons are indicated by brackets. 1: CTn1; 2: CTn2; 3: CTn3, also known as Tn*5397*; 4: CTn 4; 5: CTn5; 6: CTn6; 7:CTn7.

**Fig 10:**
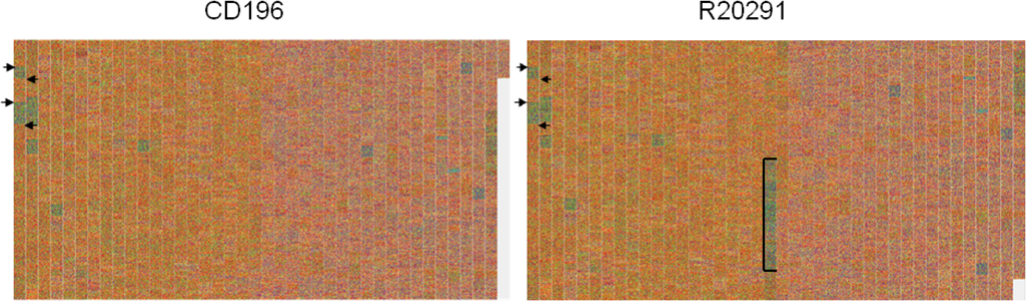
Comparison of the chromosomes of *Clostridium difficile* strains CD196 [GenBank: NC_013315] and R20291 [GenBank: NC_013316]. The region comprising transposons Tn*6104*, Tn*6105* and Tn*6106* is indicated by brackets. The two first corresponding RNA gene clusters are indicated by arrows for both strains.

**Fig 11:**
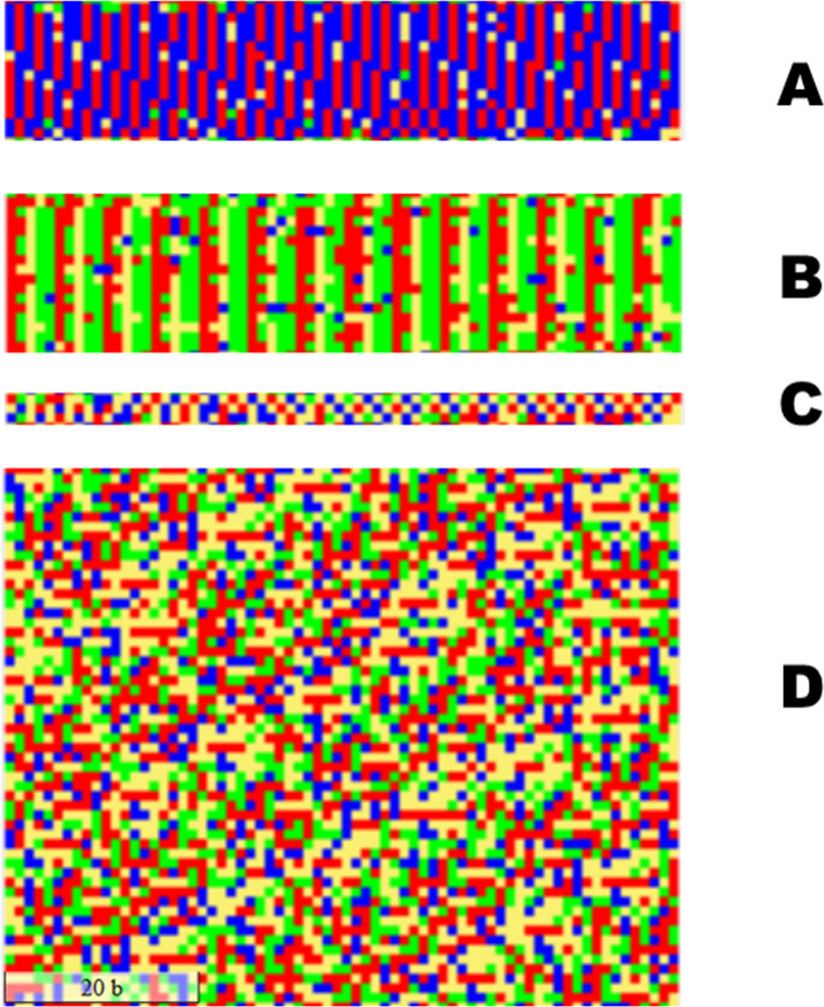
Examples of visualizations of tandem repeats in human chromosome 21 [GenBank: NC_000021.9]. The scale represents a segment of 20 nucleotides. A: microsatellite consisting of CA repeats (nucleotide coordinates 6565371–6566350); B: GGAAT tandem repeats, also known as DNA satellite III (7259491–7260680); C: imperfect [TCTA][TCTG] 4bp repeat in the D21S11 locus included in CODIS database (19181961–19182170). D: 171-base pairs repeat, also known as alphoid DNA satellite or α-satellite (7265231–7269080).

The DNA Data Visualization generator (DDV), as well as its source code, are available for download. All of the visualizations generated with this software and discussed below are also accessible with a web browser. As discussed below, the visualization method presented in this work allows immediate identification and observation of several types of sequence patterns in genomes of various sizes and origins, often not easily identified by other methods. The GenBank sequence files used to generate visualizations presented on Figs 4 and 6–11, along with URLs of the corresponding generated visualizations are listed in Table 1.

**Table 1:** Source data files used to generate visualizations presented in Figs 4 and 6–11, along with URLs of the corresponding generated visualizations.

Figure	GenBank source FASTA file	Sequence Reference	Visualization
4	http://www.ncbi.nlm.nih.gov/nuccore/86738724?report=fasta	[GenBank: NC_007777]	http://www.photomedia.ca/DDV/dnadata/nuccore86738724/
6	http://www.ncbi.nlm.nih.gov/nuccore/15594346?report=fasta	[GenBank: NC_001318]	http://www.photomedia.ca/DDV/dnadata/nuccore15594346/
7	http://www.ncbi.nlm.nih.gov/nuccore/32141095?report=fasta	[GenBank: NC_003888]	http://www.photomedia.ca/DDV/dnadata/nuccore32141095/
8	http://www.ncbi.nlm.nih.gov/nuccore/408526205?report=fasta	[GenBank: HE971709]	http://www.photomedia.ca/DDV/dnadata/nuccore408526205/
9	http://www.ncbi.nlm.nih.gov/nuccore/126697566?report=fasta	[GenBank: NC_009089]	http://www.photomedia.ca/DDV/dnadata/nuccore126697566/
10	http://www.ncbi.nlm.nih.gov/nuccore/260681769?report=fasta	[GenBank: NC_013315]	http://www.photomedia.ca/DDV/dnadata/nuccore260681769/
10	http://www.ncbi.nlm.nih.gov/nuccore/260685375?report=fasta	[GenBank: NC_013316]	http://www.photomedia.ca/DDV/dnadata/nuccore260685375/
11	http://www.ncbi.nlm.nih.gov/nuccore/224589813?report=fasta	[GenBank: NC_000021.9]	http://www.photomedia.ca/DDV/dnadata/nuccore568815577/

### Compositional asymmetry of DNA strands

Many bacterial genomes show a deviation from the classical base composition in DNA strands, [A] = [T] and [G] = [C], in a DNA replication-dependent way. Thus, G > C bias is observed in the leading strand which is replicated co-directionally with the replication fork, while C > G bias is observed in the lagging strand [14]. The bias is changing at the points of origin and termination of the DNA replication. A visually expressive example of this tendency is the main component of the genome of *Borrelia burgdorferi* [GenBank: NC_001318], the Lyme disease causing agent. It consists of a linear chromosome of almost 1 M base pairs [15]. This chromosome is replicated bi-directionally from an origin localized close to the center. The visualization of the chromosome by our method allows immediate identification of the position of the putative origin of replication on the chromosome in the segment between 458 200 and 458 400 (A and B in Fig 6). Using the tools provided with DDV software, calculation of base frequency showed that both the left and the right part of the genome have almost identical values for [A+T] (71%) and [G+C] (29%). However the GC skew, estimated from the ratio (C-G)/(C+G), as suggested by Lobry [16], switches from positive (0.211) to negative (-0.217) when calculated for 10kb window localized, respectively, upstream and downstream from the putative origin of replication (B in Fig 6), confirming that the position of the origin of replication has been correctly identified.

### Ribosomal RNA gene clusters in G+C-rich actinobacterial genomes

Actinobacteria form a branch of Gram+ bacteria with G+C-rich genomes. As a consequence, the coding sequences of protein-encoding genes show a particular codon usage pattern maximizing the use of G or C in the third position of degenerate triplets. In some genes, the third codon position G+C content can reach 98.3% [17, 18]. However, due to functional constraints, the ribosomal RNA gene clusters are relatively A+T-rich [19, 20]. The genome of the model actinomycete, *Streptomyces coelicolor* A3(2) [GenBank: NC_003888], includes six ribosomal RNA gene clusters, named *rrnA–F* [21]. All these clusters are clearly distinguishable on our visualization and two of them are shown on Fig 7. While the overall G+C ratio of the *S*. *coelicolor* genome is of 72.1%, it decreases to about 57% in rRNA gene clusters. All these clusters respect the rule saying that highly expressed genes are transcribed in the same direction as the movement of the DNA replication fork of the chromosome [22, 23]. Accordingly, a difference in GC skew is observed depending on the localization of the rRNA gene clusters relative to the DNA replication origin (~4 271 kb from the left end of the chromosome). While the global A+T and G+C proportions are similar for both clusters shown on Fig 7 (42.5% and 57.5% respectively), the (C-G)/(C+G) ratio is positive for *rrnB* but negative for *rrnA*, reflecting their respective positions on both sides of the origin of replication. On the visualization, this is translated into more abundant blue pixels for *rrnB* and green pixels for *rrnA*.

### Long terminal inverted repeats in linear chromosomes

Bacterial linear plasmids and chromosomes often include inverted repeat sequences at their ends, sometimes longer than 1 000 kb [24]. They are typically present in chromosomes of the members of the order *Actinomycetales* [14, 18, 21, 24, 25]. As an example, the recently sequenced genome of *Streptomyces davawensis* JCM 4913 [GenBank: HE971709] has long terminal inverted repeats (LTIRs) of 33.3 kb each [26]. While the G+C content of the LTIRs (69.0%) is similar to that of the entire chromosome (70.6%), the respective G and C density varies along the LTIR, what is reflected by locally more dense blue or green color on the visualization (Fig 8). Furthermore, when showed side-by-side with one segment rotated at 180° to the other, the visualized LTIRs reveal well aligned areas corresponding to tracts of higher density of complementary nucleotides (blue as opposed to green; Fig 8), as expected for inverted repeat sequences.

### Horizontal gene transfer events

Bacterial genome evolution is extensively driven by horizontal gene transfer [27]. Large DNA fragments (up to 600kb) can be acquired by conjugation, transformation and transduction and integrated into the native chromosome. This foreign DNA typically has a G+C composition that is different from the host chromosome. Therefore, such mobile elements can be discovered using our method by examining a single bacterial chromosome. As an example, *C*. *difficile* 630 [GenBank: NC_009089] (G+C ratio of 29.1%), the leading cause of hospital-acquired diarrhea, harbors seven conjugative transposons (G+C ratio of 32.7 to 42.3%) [28, 29, 30]. All seven mobile elements were identified at first sight with accuracy (rarely more than one CDS apart) (Fig 9). However, other mobile elements such as the likely mobilizable transposon Tn*5398* and prophages 1 and 2 were not identified owing to their G+C content similar to the chromosome [28, 31]. Moreover, comparison of two bacterial chromosomes allows for rapid identification of recent mobile element acquisition events. Visualization of the two closely related “hypervirulent” ribotype 027 *C*. *difficile* strains CD196 [GenBank: NC_013315] and R20291 [GenBank: NC_013316] (Fig 10) reveals the acquisition of the three transposon Tn*6104*, Tn*6105* and Tn*6106* carried on the conjugative transposon Tn*6103* in R20291, as previously reported [29].

Finally, echoing the observations aforementioned, ribosomal RNA gene clusters are also visible, because of their relatively high G+C ratio (~50%) compared to the rest of the entire chromosome (29.1%) (Fig 10).

### Human chromosomes

The 22 human autosomes as well as the two sexual chromosomes X and Y have been visualized using our method, demonstrating the scalability of the method to very large data sets. The black segments correspond mainly to heterochromatic areas of the chromosomes that are occupied by extensive tandem repeats where sequence has not been determined in detail [32] and, at least at present, constitute gaps in the genomic sequence. The determination of the sequences covered by the gaps is an active area of research [33].

It was immediately apparent that this visualization method makes it relatively easy to quickly identify regions of variable nucleotide composition density. As the image is zoomed out, the nucleotide composition information which is encoded as color is compressed through graphical algorithms that scale down images by averaging the pixel color by considering neighboring pixels. The result is that areas of high G+C concentration become even more apparent, as a combination of blue, green and cyan (sum of the two) as the user zooms out. In a similar way, the higher concentrations of A+T appear as red, yellow and orange respectively.

Tandem repeats can also be observed. Yoshoida *et al*. [4] found that the width of the visualization column does not have to be the same as the period of the repeat, an observation which is confirmed in our visualization of human chromosomes. Repeats of variable width are visible to the eye even when their period does not match the set width of 70 nucleotides per column in our visualization. Examples of dinucleotidic (A), pentanucleotidic (B) and 171bp-long repeats (D) are shown on Fig 11. Visualization of the entire chromosome was based on the genomic contig GRCh38 primary assembly [GenBank:NC_000021.9]. Areas with tandem repeats were visually identified by their regular patterns, extracted from the genomic sequence and analyzed with the Tandem Repeats Finder program [34]. The imperfect 4bp repeats (C in Fig 11) represent the D21S11 locus included in the CODIS database for forensic applications [35].

### Future Development

There are many web services available online that could be integrated with DDV, such as National Library of Medicine’s BLAST web service [36]. The requirement that FASTA files accepted by DDV have 70 nucleotides per line stems from the fact that this is the common and default FASTA format returned by NCBI’s eFetch. This requirement will be broadened to accept more formats with future development of the FASTA parser functionality in DDV. Leveraging the integration of an additional C# bioinformatics library such as .NET Bio [37] into DDV is a promising development strategy that could be used for this purpose.

DDV currently requires Windows operating system for generating visualizations, so the development of Unix and Mac OS versions of the generating software are among current development plans. Microsoft recently announced the release of .NET core as open source and cross platform [38], which simplifies the porting of DDV to other platforms and its long term sustainability. DDV is itself free and open source, and it is dependent almost entirely on open source components. DDV uses the DeepZoomTools.dll that is redistributable, but currently not open source. However, while DDV is ported to other operating systems in the future, this dll can be replaced with one of the alternative tools for the creation of DZI images, such as the free open source VIPS [39].

## Conclusions

We present a novel method for generating visual representations of nucleotide sequences. The method presented is especially practical for visualizing and navigating the DNA sequence data of whole genomes or chromosomes. We confirmed that the visualizations generated allow for the immediate identification and observation of several types of sequence patterns. This software is capable of generating interactive graphical representations of large nucleotide sequence data sets that are accessible through a web browser. In generating the visualization of *H*. *sapiens’* DNA data, we have also shown that this method scales to large data sets.

## Availability and Requirements

- **Project name:** DNA Data Visualization (DDV)
- **Project home page:** http://www.photomedia.ca/DDV/
- **Source code:** https://github.com/photomedia/DDV
- **Generated interfaces dataset:** http://dx.doi.org/10.5281/zenodo.33608
- **Operating system(s):** Windows
- **Programming languages:** C#, JavaScript, PHP
- **Other requirements:** .NET Framework 4.0 or higher
- **License:** BSD 3-Clause https://github.com/photomedia/DDV/blob/master/DDV-license.txt
- **Any restrictions to use by non-academics:** none

Producing visualizations with DDV requires a Windows operating system with .NET Framework version 4 or higher. However, any modern browser that is capable of supporting JavaScript is sufficient for end users to access and use the generated visualizations. This was tested on various browsers, including Safari (tested on version 6), Chrome (tested on version 42), Firefox (tested on version 18 and higher), Internet Explorer (tested on version 8 and 9); running on different operating systems such as Mac OS X, Windows Vista, Windows 7, Windows 8, Ubuntu and Android.
